# Prognostic Signature and Therapeutic Value Based on Membrane Lipid Biosynthesis-Related Genes in Breast Cancer

**DOI:** 10.1155/2022/7204415

**Published:** 2022-08-25

**Authors:** Yingkun Xu, Yudi Jin, Shun Gao, Yuan Wang, Chi Qu, Yinan Wu, Nan Ding, Yuran Dai, Linshan Jiang, Shengchun Liu

**Affiliations:** ^1^Department of Breast and Thyroid Surgery, The First Affiliated Hospital of Chongqing Medical University, Chongqing 400042, China; ^2^Department of Pathology, Chongqing University Cancer Hospital, Chongqing 400045, China

## Abstract

There is a need to improve diagnostic and therapeutic approaches to enhance the prognosis of breast cancer, the most common malignancy worldwide. Membrane lipid biosynthesis is a hot biological pathway in current cancer research. It is unclear whether membrane lipid biosynthesis is involved in the prognosis of BRCA. With LASSO regression, a 14-gene prediction model was constructed using data from the TCGA-BRCA cohort. The prediction model includes GPAA1, PIGF, ST3GAL1, ST6GALNAC4, PLPP2, ELOVL1, HACD1, SGPP1, PRKD2, VAPB, CERS2, SGMS2, ALDH3B2, and HACD3. BRCA patients from the TCGA-BRCA cohort were divided into two risk subgroups based on the model. Kaplan–Meier survival curves showed that patients with lower risk scores had significantly improved overall survival (*P*=2.49*e* − 09). In addition, risk score, age, stage, and TNM classification were used to predict mortality in BRCA patients. In addition, the 14 genes in the risk model were analyzed for gene variation, methylation level, drug sensitivity, and immune cell infiltration, and the miRNA-mRNA network was constructed. Afterward, the THPA website then analyzed the protein expression of 14 of these risk model genes in normal and pathological BRCA tissues. In conclusion, the membrane lipid biosynthesis-related risk model and nomogram can be used to predict BRCA clinical prognosis.

## 1. Introduction

Breast cancer is the most common cancer in women and one of the leading causes of cancer death [1–3]. Breast cancer mainly refers to malignant tumors in breast epithelial tissue, with a high clinical cure rate early. In contrast, advanced breast cancer is highly aggressive, malignant, and has sizeable histological heterogeneity [4, 5]. In the actual diagnosis and treatment process, the clinical symptoms of early breast cancer are not obvious, which is easy to cause misdiagnosis and missed diagnosis, leading to breast cancer patients missing the best time for treatment [6, 7]. At the same time, there is still no effective treatment for breast cancer patients with metastases [8]. With the continuous development of molecular biology, gene therapy has become a new direction in breast cancer treatment. However, breast cancer is a complex disease, and its occurrence and development are affected by many molecular factors. Therefore, studying the pathogenesis of breast cancer is of great significance to its treatment.

Cancer cells, as mutated cells, are the source of cancer. Unlike normal cells, cancer cells have three characteristics of infinite proliferation, transformation, and easy transfer [9]. They can proliferate indefinitely and destroy normal cells and tissues. In addition to dividing out of control, cancer cells can invade surrounding normal tissues locally and even metastasize to other body parts via the circulatory system or lymphatic system. By remodeling their metabolism, cancer cells provide ATP and macromolecules necessary for cell growth, division, and survival. To meet their rapid proliferation needs, tumor cells exhibit metabolic characteristics different from normal tissue cells [10, 11]. Although cancer types and etiologies are different, there are certain similarities in the changes in metabolic pathways in cancer cells. Glucose, pentose phosphate, and glutamine metabolic pathways adapt to cancer cells' rapid growth and proliferation. Recent studies have shown that membrane lipid biosynthesis is also altered in cancer cells [12–14]. In cancer development, changes in membrane lipid biosynthesis play an essential role in providing biomembrane macromolecules and signaling molecules for the occurrence and development of cancer [15–18]. Alterations in membrane lipid biosynthesis in tumor tissues provide macromolecular precursors necessary for their abnormal survival and growth. This suggests that clinical actions targeting this critical metabolism could be a highly effective therapeutic approach [19–23].

As a computer science and high-throughput sequencing technology developed, bioinformatic analysis can provide researchers with more opportunities to explore the genetic risk, regulatory mechanism, and protein function of cancer. As a highly representative bioinformatics database, the TCGA database is generated by large-scale gene sequencing, which can quantitatively study and analyze the changes in gene expression during tumor occurrence and development [24, 25]. Therefore, in this study, we performed a series of in-depth explorations of membrane lipid biosynthesis in BRCA based on the TCGA database using multiple bioinformatic analyses. Our findings suggest that targeting membrane lipid biosynthesis has excellent potential for BRCA prevention, treatment, and control.

## 2. Materials and Methods

### 2.1. Data Acquisition and Selection of Membrane Lipid Biosynthesis-Related Genes

This study downloaded BRCA mRNA expression and clinical information from TCGA (https://portal.gdc.cancer.gov/). A total of 1,109 tumor samples and 113 normal samples with clinical and expression data were obtained. Expression data were normalized using log_2_ (TPM + 1) transformation. Transcript data and human profiles are matched and sorted by Perl to get comprehensive and accurate mRNA gene expression data using information from an integrated database. Gene IDs are converted to gene names for subsequent analysis. Membrane lipid biosynthesis-related genes were obtained from the Gene Set Enrichment Analysis (GSEA) database (https://www.gsea-msigdb.org/gsea/index.jsp) [26, 27]. The standard name is GOBP membrane lipid biosynthetic process. The systematic name is M16158. Differential expression analysis was carried out on TCGA data using the “limma” *R* package. Univariate Cox analysis was used to identify membrane lipid biosynthesis-related genes with prognostic values (*P* < 0.05). The TCGA cohort was evaluated for membrane lipid biosynthesis-related genes with predictive value.

### 2.2. Construction and Validation of a Prognostic Signature of Membrane Lipid Biosynthesis-Related Genes

A predictive model was constructed in BRCA using LASSO regression analysis to minimize the use of overlapping membrane lipid biosynthesis-related gene risk values. Statistics and machine learning use LASSO regression to identify the most critical factors and improve the accuracy of statistical models. LASSO is a popular machine learning algorithm widely used in medical research [25, 28, 29]. In our study, risk scores were obtained for all patients and then divided into high- or low-risk subgroups based on the median risk score of the TCGA cohort. The “survival,” “survminer,” and “timeROC” packages in *R* were used to perform KM survival analysis and ROC analysis based on OS to estimate the predictive accuracy of the two sets of gene signatures.

### 2.3. Prognostic Independence of Prognostic Signature from Routine Clinicopathological Features and Generation of the Corresponding Nomogram

To further evaluate the gene prognostic signature's independent predictive value, it was examined using univariate and multivariate Cox regression analysis to see if it was affected by other clinical traits such as age, gender, stage, and TNM stage. Corresponding nomograms were drawn using the *R* package “RMS” based on risk scores and other routine clinicopathological features.

### 2.4. Prognostic Value and Gene Variation of Risk Model Genes Associated with Membrane Lipid Biosynthesis in Pan-Cancer

In this study, we first targeted the membrane lipid biosynthesis-related risk model genes GPAA1, PIGF, ST3GAL1, ST6GALNAC4, PLPP2, ELOVL1, HACD1, SGPP1, PRKD2, VAPB, CERS2, SGMS2, ALDH3B2, and HACD3 were analyzed for OS, CNV, SNV, methylation, and immune cell infiltration in pan-cancer via Gene Set Cancer Analysis (GSCA) database (https://bioinfo.life.hust.edu.cn/GSCA/#/) [30]. We then used this database to drill down to the percentage of cancers, in which mRNA expression of membrane lipid biosynthesis-related risk model genes had a potential impact (activation or inhibition) on biological pathways. Finally, we used this database to construct a miRNA regulatory network for membrane lipid biosynthesis-related risk model genes.

### 2.5. Anticancer Drug Sensitivity Analysis of Risk Model Genes Associated with Membrane Lipid Biosynthesis

This study integrated the drug sensitivity and gene expression profiling data from Genomics of Drug Sensitivity in Cancer (GDSC) and Cancer Therapeutics Response Portal (CTRP). The correlation between small molecule/drug sensitivity (IC50) and the expression of 14 membrane lipid biosynthesis-related risk model genes was analyzed by Spearman correlation. GDSC was developed by the Sanger Institute in the United Kingdom to collect the sensitivity and response of tumor cells to drugs. Raw data from GDSC comes from 75,000 experiments describing the response of about 200 anticancer drugs in more than 1,000 tumor cells. The oncogene mutation information in this database comes from the COSMIC database, including oncogene point mutations, gene amplification and loss, tissue types, and expression profiles (https://www.cancerrxgene.org/) [31–33]. In addition, CTRP, a database of 70,000 cancer cell line compounds that link susceptibility to genetic or lineage characteristics, serves as a public resource for researchers worldwide (https://portals.broadinstitute.org/ctrp.v2.1/) [34–36]. This important resource drives the discovery of potential cancer drugs that match the patient population and maximize the likelihood that patients will benefit from them. Such data are therefore crucial for the discovery of potential tumor therapeutic targets.

### 2.6. Gene Set Enrichment Analysis and Protein Expression Analysis of Membrane Lipid Biosynthesis-Related Risk Model Genes in BRCA

Gene Set Enrichment Analysis (GSEA) is a method for enrichment analysis of target genes, which can be used to test the correlation between target genes and gene sets of known functions. This study used the GTBA database to explore the relationship between membrane lipid biosynthesis-related risk model genes and the HALLMARK pathway (https://guotosky.vip:13838/GTBA/). The Human Protein Atlas (THPA) database is based on proteomics, transcriptomics, and systems biology data, which can map tissues, cells, organs, etc. This database includes protein expression in tumors and normal tissues (https://www.proteinatlas.org/) [37, 38]. Therefore, in this study, we used this database to explore the expression of risk model genes related to membrane lipid synthesis in breast cancer and normal breast tissue.

### 2.7. Tumor Immune Estimation Resource (TIMER) Database

A comprehensive database of immune infiltration in various cancer types, the TIMER database offers multiple methods of assessing immune infiltration levels, providing researchers with the ability to generate high-quality graphs online to explore immunomics, clinical manifestations, and tumor genomes. This study used this database to assess the association between membrane lipid biosynthesis-related risk model genes and immune cell infiltration in BRCA.

### 2.8. Statistical Analysis

Statistical analysis was performed using *R* 4.1.2 and Strawberry Perl software. The non-parametric rank test (Wilcox test) was used to analyze the expression differences of membrane lipid biosynthesis-related genes in breast cancer and normal breast tissue samples. The Kaplan–Meier analysis of gene expression was used to analyze the relationship between breast cancer survival and gene expression, and the significance of the breast cancer risk model was assessed using univariate and multivariate Cox regression analysis. The relationship between gene expression and clinicopathological characteristics was analyzed by *χ*^2^ test.

## 3. Results

### 3.1. Gene Expression and Protein Interaction of Membrane Lipid Biosynthesis-Related Molecules in BRCA

**Figure 1:**
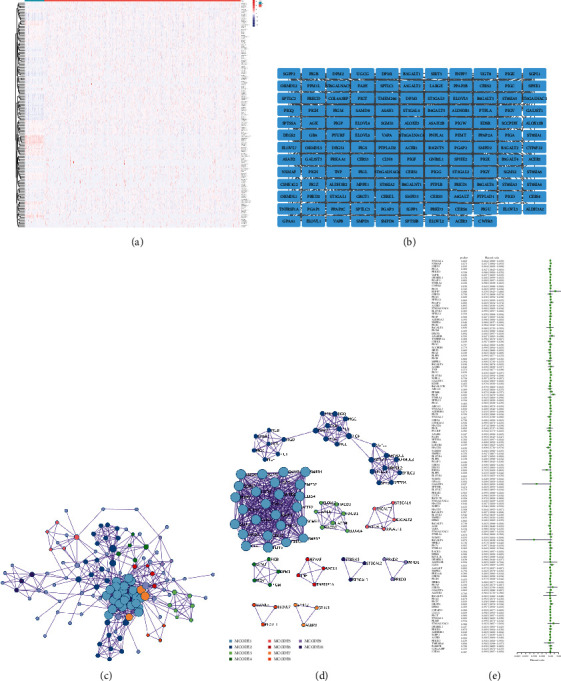
Expression and interaction of membrane lipid biosynthesis-related molecules in BRCA. (a) Heat map showing the mRNA expression of membrane lipid biosynthesis-related genes in BRCA. (b) Protein-protein interaction network showing interactions of proteins encoded by genes involved in membrane lipid biosynthesis. (c, d) Protein interaction network diagrams show clustering analysis results based on the MCODE algorithm. (e) The forest plot shows the univariate Cox analysis of membrane lipid biosynthesis-related genes in BRCA. ^*∗*^*P* < 0.05, ^*∗∗*^*P* < 0.01, and ^*∗∗∗*^*P* < 0.001.

It is well known that the process of carcinogenesis is often accompanied by abnormal expression of gene mRNA. These abnormally expressed genes are of more interest to cancer researchers than those that are not [9, 39]. To explore whether there are differences in the expression of membrane lipid biosynthesis-related genes in BRCA, we extracted the mRNA expression data of membrane lipid biosynthesis-related genes from the TCGA database. We used the *R* language to draw a corresponding heatmap (Figure 1(a)). We can intuitively see that the mRNA expression of the vast majority of membrane lipid biosynthesis-related genes is significantly different in BRCA. Then, to explore the interaction of the protein molecules encoded by membrane lipid biosynthesis-related genes, we used the String website to draw the corresponding PPI network (Figure 1(b)). The PPI network revealed extensive interactions of membrane lipid biosynthesis-related molecules. To further explore the relationship, we used the MCODE algorithm of the Metascape website to perform cluster analysis on the PPI network to identify subnetworks, that is, potential protein complexes (Figures 1(c) and 1(d)) (Supplementary Material Table S1). The results showed that the top three subnetworks were sphingolipid metabolism, membrane lipid metabolic process, and fatty acid elongation. Finally, the results of univariate Cox regression analysis showed that GPAA1, PIGF, ST3GAL1, ST6GALNAC4, PLPP2, ELOVL1, PIGU, VAPB, CERS2, SGMS2, ALDH3B2, and HACD3 play an inhibitory role in the malignant progression of BRCA, in contrast, HACD1, SGPP1, and PRKD2 play an inhibitory role in BRCA malignant progression (Figure 1(e)).

### 3.2. Construction of Risk Models Associated with Membrane Lipid Biosynthesis in BRCA

**Figure 2:**
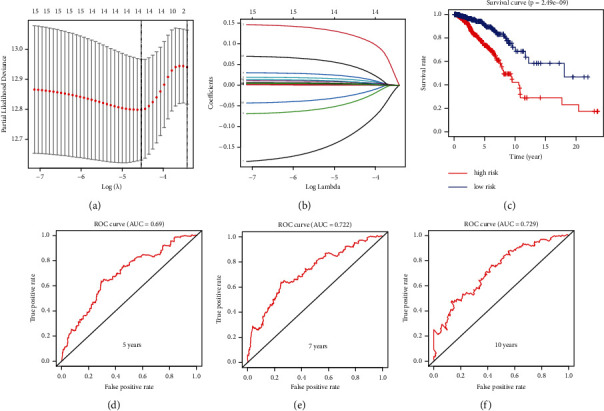
Construction of risk models using membrane lipid biosynthesis-related prognostic genes in BRCA. (a) LASSO regression of OS-related genes. (b) Cross‐validation for tuning the parameter selection. (c) Based on this membrane lipid biosynthesis-related risk model, the Kaplan–Meier curve in BRCA showed that the overall survival rate of the low-risk group was significantly higher than that of the high-risk group (*P*=2.49*e* − 09). Among them, blue represents the low-risk group and red represents the high-risk group. (d–f) The AUC of the prediction of 5-, 7-, and 10‐year survival rates of BRCA.

To fully explore the prognostic value of membrane lipid biosynthesis-related genes in BRCA, we used LASSO regression curve analysis to establish a prognostic risk model in BRCA, which included GPAA1, PIGF, ST3GAL1, ST6GALNAC4, PLPP2, ELOVL1, HACD1, SGPP1, PRKD2, VAPB, CERS2, SGMS2, ALDH3B2, and HACD3 (Figures 2(a) and 2(b)). LASSO regression curve analysis is often used in medical modeling [28, 29]. Based on the calculation formula of the risk model related to membrane lipid biosynthesis, we can divide breast cancer patients into high-risk and low-risk groups. The drawn survival curve shows that the survival rate of breast cancer patients in the high-risk group is significantly lower than that in the low-risk group. cancer patients (*P*=2.49*e* − 09) (Figure 2(c)). To test the predictive accuracy of this model of membrane lipid biosynthesis-related risk, we performed a ROC curve analysis, which showed that the five-year AUC was equal to 0.69, the seven-year AUC was equal to 0.722, and the ten-year AUC was equal to 0.729 (Figures 2(d)–2(f)). This indicates that this membrane lipid biosynthesis-related risk model has good predictive accuracy. The formula for calculating the risk model is as follows.

Risk model = 0.004049719 *∗* GPAA1 + 0.119845071 *∗* PIGF + 0.002817462 *∗* ST3GAL1 + 0.019867802 *∗* ST6GALNAC4 + 0.013021966 *∗* PLPP2 + 0.007145373 *∗* ELOVL1 + 0.00779386 *∗* VAPB + 0.000841801 *∗* CERS2 + 0.045125517 *∗* SGMS2 + 0.001158905 *∗* ALDH3B2 + 0.002111798 *∗* HACD3 − 0.116447762 *∗* HACD1 − 0.040517734 *∗* SGPP1 − 0.023673124 *∗* PRKD2.

### 3.3. Independent Predictive Value of Risk Models Associated with Membrane Lipid Biosynthesis and Construction of Corresponding Predictive Survival Nomogram in BRCA

**Figure 3:**
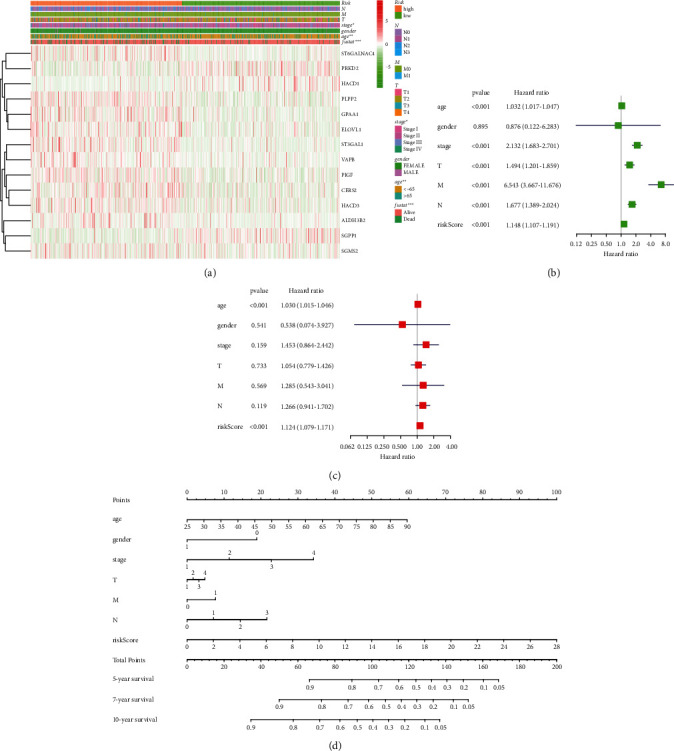
Based on this risk model associated with membrane lipid biosynthesis, univariate and multivariate Cox regression curve analysis was performed and a corresponding nomogram was generated in BRCA. (a) Heatmap showing correlations between membrane lipid biosynthesis-related risk models and clinicopathological features of breast cancer patients. (b, c) Forest plots showing the results of univariate Cox analysis and multivariate Cox analysis. (d) Based on this membrane lipid biosynthesis-related risk model, a corresponding nomogram was drawn to predict breast cancer patients' 5-, 7-, and 10-year survival.

To gain an in-depth understanding of the correlation between membrane lipid biosynthesis-related risk models and clinical clinicopathological characteristics, we obtained the corresponding BRCA clinical data through the TCGA official website and performed a correlation analysis. The results are presented in the form of a heatmap. The results showed a significant correlation between this membrane lipid biosynthesis-related risk model and stage, age, and fustat in breast cancer patients (Figure 3(a)). We subsequently performed univariate and multivariate Cox regression analyses to explore the role of this membrane lipid biosynthesis-related risk model and clinicopathological features in breast cancer progression. Univariate Cox regression analysis showed that age, stage, *T*, *M*, *N*, and risk score were risk factors for the malignant progression of breast cancer (Figure 3(b)). Multivariate Cox regression analysis showed that age and risk score were independent risk factors for the malignant progression of breast cancer (Figure 3(c)). Finally, based on the risk model associated with membrane lipid biosynthesis, we drew corresponding nomograms to predict breast cancer patients 5-, 7-, and 10-year survival (Figure 3(d)). The above results indicate that this membrane lipid biosynthesis-related risk model has solid clinical relevance and clinical application value in BRCA.

### 3.4. Gene Expression and Prognostic Value of Risk Model Genes Associated with Membrane Lipid Biosynthesis in Pan-Cancer

**Figure 4:**
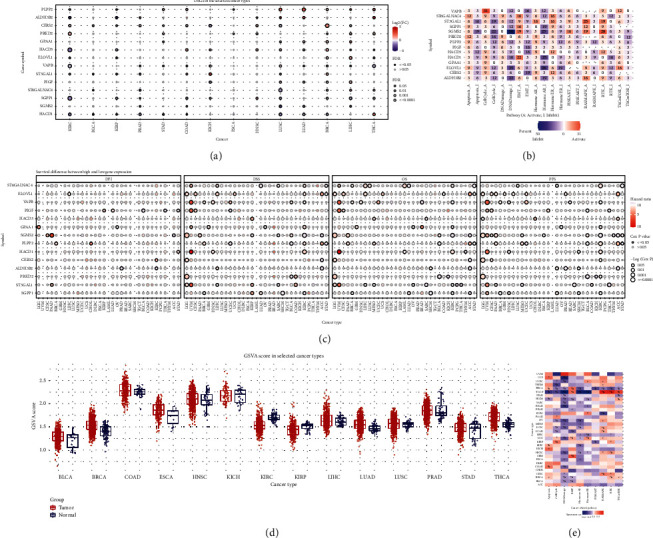
Differences in expression, prognosis, and biological pathway correlation of risk model genes associated with membrane lipid biosynthesis in pan-cancer. (a)–(b) Heatmaps showing differential mRNA expression of these membrane lipid biosynthesis-related risk model genes in pan-cancer and their correlation with biological pathways. (c) Heat map showing DFI, DSS, OS, and PFS of these membrane lipid biosynthesis-related risk model genes in pan-cancer. (d, e) These show how the GSVA scores of these membrane lipid biosynthesis-related risk model genes differ in pan-cancer and their correlation with biological pathways. The red represents the activated biological pathway, and the blue represents the inhibited biological pathway.

To further understand the significance of membrane lipid biosynthesis-related risk model genes in pan-cancer, first, we explored the mRNA expression differences of these fourteen risk model genes in pan-cancer and presented them in the form of a heat map. The results showed that these fourteen risk model genes were widely differentially expressed in LUSC, LUAD, BRCA, LIHC, and THCA (Figure 4(a)). Subsequently, we performed a biological pathway correlation analysis for these fourteen risk model genes. The results showed that its mRNA expression was negatively correlated with the inhibition of DNA Damage, EMT, and Hormone AR pathways. In contrast, its mRNA expression was positively associated with activation of Apoptosis, Cell Cycle, and EMT pathways (Figure 4(b)). Furthermore, to closely link the clinic, we explored the predictive value of risk model genes associated with membrane lipid biosynthesis in pan-cancer. The results showed that these fourteen risk model genes were significantly associated with DSS, OS, and PFS in patients with multiple types of cancer (Figure 4(c)). We then performed a Gene set variation analysis (GSVA) on these fourteen risk model genes to explore the differences in multiple tumors of the membrane lipid biosynthesis-related risk model genes. GSVA is a non-parametric, unsupervised algorithm that transforms gene expression data, from an expression matrix featuring a single gene to an expression matrix featuring a specific set of genes. GSVA quantified gene enrichment results, making the subsequent statistical analysis more convenient. Boxplots show that the GSVA scores of these fourteen risk model genes tended to increase in various tumors, including BLCA, BRCA, ESCA, LUAD, and THCA (Figure 4(d)). The biological pathway correlation analysis showed a negative correlation between GSVA score and DNA Damage (Figure 4(e)). The above results suggest that this membrane lipid biosynthesis-related risk model gene may play a similar biological function in pan-cancer and provide meaningful clues for future cancer research.

### 3.5. Genetic Variation of Risk Model Genes Associated with Membrane Lipid Biosynthesis in Pan-Cancer

**Figure 5:**
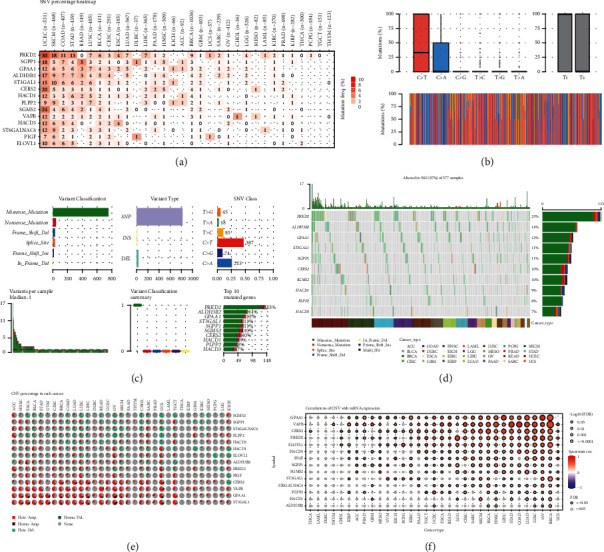
Variation of risk model genes associated with membrane lipid biosynthesis in pan-cancer. (a–d) These show the SNVs of these membrane lipid biosynthesis-related risk model genes in pan-cancer. (e) Heatmap showing the CNVs of these membrane lipid biosynthesis-related risk model genes in pan-cancer. (f) Heatmap showing the correlation between CNV and mRNA expression of risk model genes.

Variations in the human genome play an essential role in inherited diseases. In addition to DNA point mutations, the genome involves variation in large DNA sequences, including microduplications and microdeletions of submicroscopic structures. Therefore, we further explored the genetic variation of risk model genes related to membrane lipid biosynthesis in pan-cancer. In the SNV variant analysis results, we found that PRKD2, SGPP1, and GPAA1 have widespread high CNVs in UCEC, SKCM, COAD, and STAD. The results also showed that the top 10 SNV variants were PRKD2, ALDH3B2, GPAA1, ST3GAL1, SGPP1, CERS2, SGMS2, HACD1, PLPP2, and HACD3. Among them, PRKD2 accounted for as high as 22% of SNVs in pan-cancer (Figures 5(a)–5(d)). Since CNVs are an essential part of genomic variation, we then explored the CNVs of membrane lipid biosynthesis-related risk model genes in pan-cancer. The results showed that CERS2, VAPB, GPAA1, and ST3GAL1 were widespread CNVs in pan-cancer (Figure 5(e)). In LUAD, LUSC, OV, BRCA, and UCS, CNVs of GPAA1, VAPB, ERS2, PRKD2, and ELOVL1 showed a positive correlation with their mRNA expression (Figure 5(f)).

### 3.6. Methylation and Drug Sensitivity of Risk Model Genes Associated with Membrane Lipid Biosynthesis in Pan-Cancer

**Figure 6:**
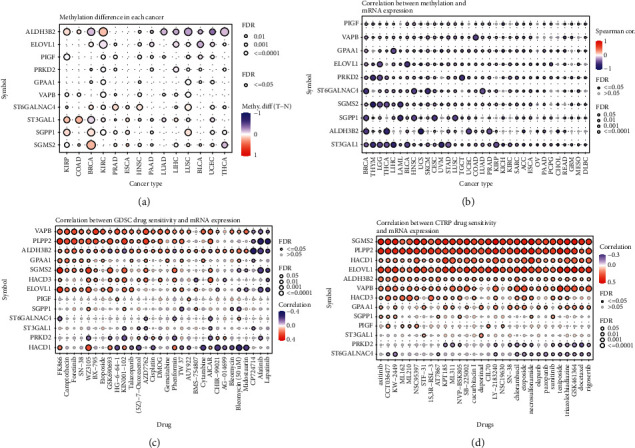
Correlations between methylation, drug sensitivity, and mRNA expression of risk model genes associated with membrane lipid biosynthesis. (a) Heatmap showing differences in methylation levels of these membrane lipid biosynthesis-related risk model genes across multiple tumors. (b) Heat map showing the correlation between methylation levels and mRNA expression of these membrane lipid biosynthesis-related risk model genes. (c, d) Heatmaps showing correlations between these membrane lipid biosynthesis-related risk model genes and susceptibility to multiple anticancer drugs in the GDSC and CTRP databases.

Epigenetic studies are the characteristics of heritable changes outside the DNA sequence. All cells of a living individual have essentially the same DNA. Still, different organs and tissues have different functions and maintain their specific cellular identity over multiple cell divisions, which are primarily thought to be determined by the appearance of mediated by genetic information [9, 40]. Since methylation is essential to epigenetic research, we explored the methylation differences of membrane lipid biosynthesis-related genes in various tumors [41, 42]. We presented them in the form of heat maps. The results showed that ALDH3B2, ST3GAL1, and SGMS2 genes had noticeable methylation differences in BRCA, KIRC, and LUSC (Figures 6(a) and 6(b)). Because mutations in the cancer genome can affect the efficacy of clinical treatments, responses to anticancer drugs vary widely across different therapeutic targets. Therefore, we used data from GDSC and CTRP databases to explore the sensitivity of membrane lipid biosynthesis-related genes to various anticancer drugs. The results showed that SGMS2, PLPP2, HACD1, ELOVL1, ALDH3B2, and VAPB significantly correlated with the sensitivity of various anticancer drugs. Meanwhile, there were significant negative correlations between PRKD2, ST6GALNAC4, and HACD1 sensitivities to multiple anticancer drugs (Figures 6(c) and 6(d)). We believe our study provides valuable data for future drug target development.

### 3.7. Immune Cell Infiltration of Membrane Lipid Biosynthesis-Related Risk Model Genes in BRCA

**Figure 7:**
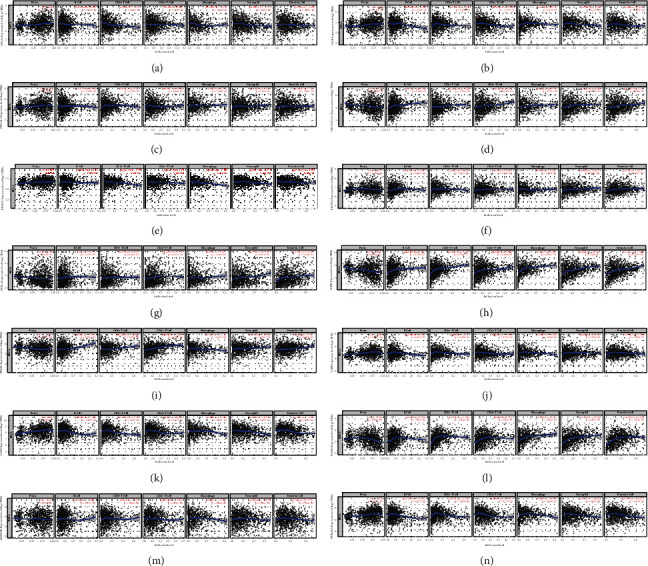
Scatter plot showing the correlation between membrane lipid biosynthesis-related risk model genes and immune cell infiltration in BRCA. (a–n) GPAA1, PIGF, ST3GAL1, ST6GALNAC4, PLPP2, ELOVL1, HACD1, SGPP1, PRKD2, VAPB, CERS2, SGMS2, ALDH3B2, and HACD3; the data come from TIMER database.

We all know that tumor tissue is not simply composed of tumor cells, it is composed of various types of cells, including stromal cells, fibroblasts, immune cells, etc. These cells constitute the tumor microenvironment [43, 44]. In recent years, we have paid more attention to the role of immune cells in this microenvironment [45–47]. Immune cells include many kinds, such as *B* cells and *T* cells. Different immune cells play different roles in the process of tumorigenesis, and the composition of immune cells of various tumors also has its characteristics. Because of the importance of immune cell infiltration in tumor progression, based on the Timer database, we examined six types of immune cell infiltration and this membrane lipid biosynthesis-related gene in BRCA. The results showed a significant positive correlation between ST3GAL1, SGPP1, VAPB, and SGMS2 and CD8+ *T* Cell infiltration (Figures 7(a)–7(n)).

### 3.8. Biological Pathway Enrichment Analysis and Protein Expression of Membrane Lipid Biosynthesis-Related Risk Model Genes in BRCA

**Figure 8:**
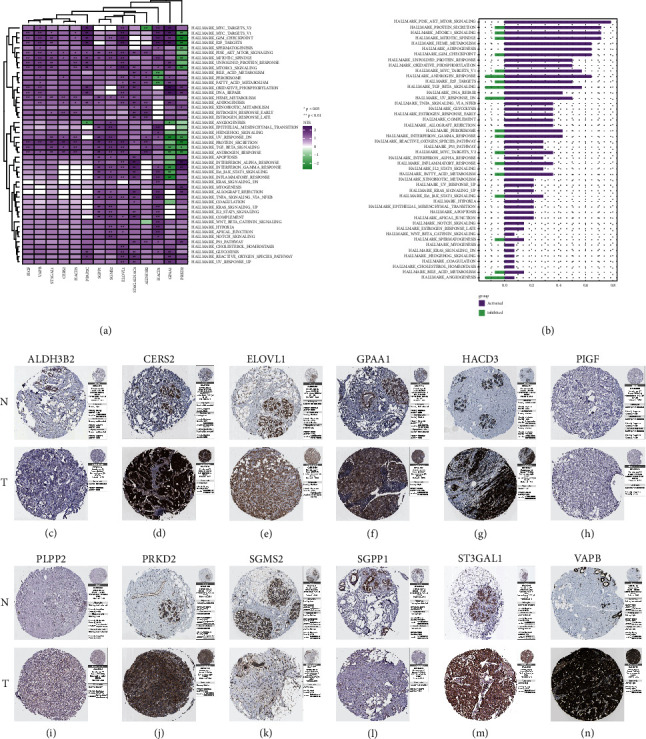
Pathway enrichment and protein expression analysis of membrane lipid biosynthesis-related risk model genes in BRCA. (a, b) These show pathway enrichment analysis in BRCA for these membrane lipid biosynthesis-related risk model genes and their corresponding quantitative histograms. (c–n) Protein expression of membrane lipid biosynthesis-related risk model genes ALDH3B2, CERS2, ELOVL1, GPAA1, HACD3, PIGF, PLPP2, PRKD2, SGMS2, SGPP1, ST3GAL1, and VAPB in breast cancer and normal breast tissue; the picture data come from THPA database.

In this study, we performed GSEA on the HALLMARK gene set for membrane lipid biosynthesis-related risk model genes in BRCA (Figures 8(a) and 8(b)) [26, 48]. GSEA results showed that these risk model genes were associated with abnormal activation of PI3K AKT MTOR SIGNALING, PROTEIN SECRETION, and MTORC1 SIGNALING, HEME METABOLISM, and OXIDATIVE PHOSPHORYLATION. Subsequently, we used immunohistochemical data from the THPA database to validate our previous findings to explore the expression of risk model genes associated with membrane lipid biosynthesis in mammary and normal breast tissues. Since ST6GALNAC4 and HACD1 have not been included in the THPA database, we provide the immunohistochemical results for ALDH3B2, CERS2, ELOVL1, GPAA1, HACD3, PIGF, PLPP2, PRKD2, SGMS2, SGPP1, ST3GAL1, and VAPB. The results showed that the protein expression levels confirmed our previous findings at the mRNA level, confirming our findings were correct (Figures 8(c)–8(n)). These data may provide a solid basis for further research into these risk model genes and pave the way for future interventions.

### 3.9. Construction of a Risk Model Genes-Related miRNA Interaction Network for Membrane Lipid Biosynthesis

**Figure 9:**
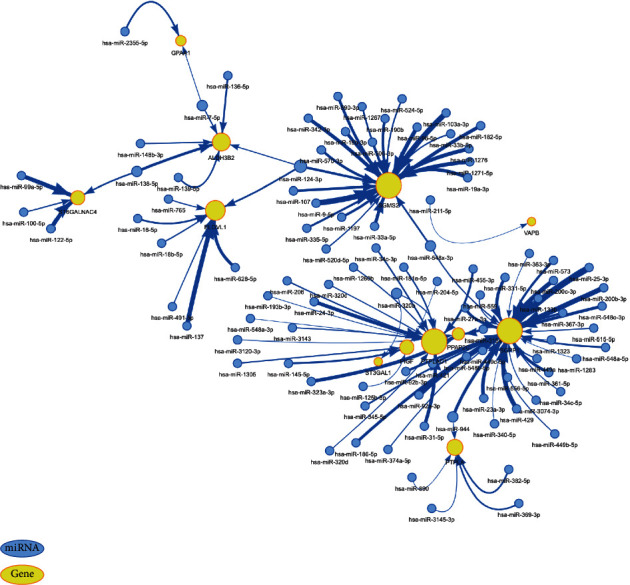
miRNA regulatory networks associated with risk model genes related to membrane lipid biosynthesis. Among them, blue represents miRNA and yellow represents genes.

MicroRNAs (miRNAs) are a class of endogenous non-coding RNAs with a length of about 22 nt, which regulate gene expression by complementary binding to target gene transcripts [49, 50]. In recent years, studies have found that miRNAs are closely related to the occurrence of cancer, and miRNAs are involved in regulating multiple aspects of tumors, including transcription, cell cycle regulation, apoptosis, angiogenesis, tumor invasion, and invasion metastasis. miRNAs can directly act as oncogenes or tumor suppressor genes to affect the occurrence and growth of tumors. To further explore the regulatory relationship between membrane lipid biosynthesis-related risk genes and miRNAs, we used the GSCALite database to map the miRNA-mRNA regulation network associated with membrane lipid biosynthesis-related risk genes (Figure 9). Among them, we can find a regulatory relationship between a variety of miRNAs and the SGMS2 gene, which suggests that we can target these miRNAs to regulate SGMS2 in the future, thereby affecting the progression of BRCA.

## 4. Discussion

Globally, cancer is the leading cause of death and a significant barrier to extending life [51]. With the aging population and the rapid development of society, the cancer burden in countries worldwide is overgrowing [52, 53]. It is worth noting that in 2020, breast cancer in women surpassed lung cancer as the most common cancer worldwide for the first time, with an estimated 2,261,419 new cases. Meanwhile, breast cancer is the fifth leading cause of cancer death globally, with 684,996 deaths. In recent years, the morbidity and mortality of female breast cancer worldwide have increased sharply, and the disease burden has also increased, becoming a significant global public health problem [54]. In recent years, the biological role of membrane lipid biosynthesis-related genes in cancer has attracted more and more attention from cancer researchers. Therefore, in this study, we used “glmnet” and “survival” extension packages to perform LASSO regression curve analysis. We utilized genes related to membrane lipid biosynthesis to construct a prognosis-related risk model in BRCA. The risk model contains fourteen genes, namely, GPAA1, PIGF, ST3GAL1, ST6GALNAC4, PLPP2, ELOVL1, HACD1, SGPP1, PRKD2, VAPB, CERS2, SGMS2, ALDH3B2, and HACD3.

GPAA1 is a crucial subunit of glycosylphosphatidylinositol transferase, responsible for binding GPI anchors to precursor proteins, but not involved in GPI anchor synthesis. Previous studies have shown that GPAA1 is abnormally expressed and amplified in various malignant tumors, such as liver, breast, gastric, and colorectal [55–58]. Since GPAA1 is an enzyme, its chemical activity is vulnerable to the intervention of various physicochemical factors or inhibitors, so the development of its inhibitors is less complex and more drug-producing. Therefore, we believe that targeting GPAA1-mediated synthesis of GPI-anchored proteins is a potential tumor-targeted therapeutic option [55]. Furthermore, PIGF is a homodimeric glycoprotein belonging to the VEGF subfamily. It exists in two isoforms, PIGF-1 and PIGF-2, the latter having a heparin-binding domain. Like VEGF, it is a potent angiogenic factor. Tumor growth promotes the proliferation of tumor blood vessels by interacting with its receptors, thereby affecting tumor growth, infiltration, and metastasis [59, 60]. And ST3GAL1 regulates cancer progression by affecting the activity of the TGF-*β* signaling pathway in glioblastoma and breast cancer [61, 62].

Additionally, SGPP1 controls multiple cellular functions, including differentiation, proliferation, metastasis, cytoskeleton remodeling, senescence, and apoptosis [63]. SGPP1 is a tumor suppressor gene that plays a crucial role in cancer invasion and metastasis [64–66]. In gastric cancer tissues, the expression of SGPP1 was down-regulated compared with adjacent tissues and cancer-free tissues. It was confirmed that the low expression of SGPP1 was positively correlated with the distant metastasis of lymph nodes and gastric cancer. SGPP1 downregulation can promote cell migration to epidermal growth factor (EGF), while SGPP1 overexpression can decrease chemotaxis to EGF [67]. There is evidence that PRKD2 regulates fundamental biological processes, including signal transduction, membrane trafficking, cell survival, migration, differentiation, and proliferation [68–71]. PRKD2 affects drug resistance in various tumors such as breast cancer and leukemia by regulating tumor cell proliferation, apoptosis, metastasis, and invasion [72]. As in glioblastoma, PRKD2 can promote CDKN1A gene overexpression through p53-dependent or independent pathways and integrated extracellular signal regulation, promoting cellular senescence and affecting cell sensitivity [73]. Furthermore, SGMS2 is a key regulator involved in ceramide and sphingomyelin homeostasis. A previous study showed that high expression of SGMS2 is associated with breast cancer metastasis. SGMS2 promotes cancer cell invasion by enhancing TGF-*β*/smad signaling to initiate epithelial-mesenchymal transition [74].

Meanwhile, several other risk model genes were identified in this study (ST6GALNAC4, PLPP2, ELOVL1, HACD1, VAPB, CERS2, ALDH3B2, and HACD3) have not yet been explored in depth in breast cancer. In the future, we should focus on these genes' biological functions and roles in the malignant progression of breast cancer. This study still has some limitations, mainly divided into the following three points. First, the data of BRCA patients used in this study were extracted from TCGA, and data from more databases are still needed for validation; second, although the newly proposed risk model has apparent clinical significance, its underlying mechanisms are still not precise. Therefore, we plan to explore further the role of this risk model in single-center or multicenter clinical samples in the future. Third, due to experimental conditions and time constraints, we have not yet validated the biological functions of risk model genes in BRCA. Therefore, we hope to plan to explore the biological processes of these risk models in BRCA through in vivo and in vitro experiments in the future.

## 5. Conclusions

This study deeply explored the potential biological functions and application values of membrane lipid biosynthesis-related genes in various tumors, including breast cancer. Surprisingly, we successfully constructed a fourteen-gene risk model in BRCA using these lipid biosynthesis-related genes. Based on this risk model, we can easily divide BRCA patients into high-risk and low-risk groups. In addition, to facilitate future practical applications, we also draw a nomogram corresponding to this risk. Therefore, we believe that this study guides the precision treatment of breast cancer by interpreting genomic data and lays the foundation for future scientific research and molecular typing of BRCA.

## Data Availability

The data used to support the findings of this study are available from the corresponding author upon request.

## Abbreviations

BRCA: Breast cancer
TCGA: The Cancer Genome Atlas
GSCA: Gene set cancer analysis
THPA: The Human Protein Atlas
ImmuCellAI: Immune cell abundance identifier
GDSC: Genomics of drug sensitivity in cancer
CTRP: Cancer therapeutics response portal
LASSO: Least absolute shrinkage and selection operator
GSEA: Gene set enrichment analysis
GPAA1: Glycosylphosphatidylinositol anchor attachment 1
PIGF: Phosphatidylinositol glycan anchor biosynthesis class F
ST3GAL1: ST3 beta-galactoside alpha-2,3-sialyltransferase 1
ST6GALNAC4: ST6 N-acetylgalactosaminide alpha-2,6-sialyltransferase 4
PLPP2: Phospholipid phosphatase 2
ELOVL1: ELOVL fatty acid elongase 1
HACD1: 3-hydroxyacyl-CoA dehydratase 1
SGPP1: Sphingosine-1-phosphate phosphatase 1
PRKD2: Protein kinase D2
VAPB: VAMP-associated proteins B and C
CERS2: Ceramide synthase 2
SGMS2: Sphingomyelin synthase 2
ALDH3B2: Aldehyde dehydrogenase 3 family member B2
HACD3: 3-hydroxyacyl-CoA dehydratase 3
EGF: Epidermal growth factor.

## Supplementary Materials

Supplementary Materials
*Table S1*. Cluster analysis results of membrane lipid biosynthesis-related genes under the MCODE algorithm.
