# Supplementary material for: Prognostic Signature and Therapeutic Value Based on Membrane Lipid Biosynthesis-Related Genes in Breast Cancer
Source: J Oncol. 2022 Aug 25;2022:7204415. doi: 10.1155/2022/7204415 (PMC9436593; doi:10.1155/2022/7204415)
Supplement: Supplementary Materials — Table S1. Cluster analysis results of membrane lipid biosynthesis-related genes under the MCODE algorithm. [file 7204415.f1.pdf]

Network	Annotation
MyList	GO:0046513|ceramide biosynthetic process|-100.0;GO:0030148|sphingolipid biosynthetic process|-100.0;<br>GO:0046467|membrane lipid biosynthetic process|-100.0
MyList_MCODE_ALL	GO:0046467|membrane lipid biosynthetic process|-100.0;GO:0030148|sphingolipid biosynthetic process|-100.0;<br>GO:0006643|membrane lipid metabolic process|-100.0
MyList_SUB1_MCODE_1	hsa00600|Sphingolipid metabolism|-71.8;R-HSA-428157|Sphingolipid metabolism|-64.7;GO:0006665|sphingolipid metabolic process|-58.0
MyList_SUB1_MCODE_2	GO:0006643|membrane lipid metabolic process|-51.4;GO:0046467|membrane lipid biosynthetic process|-48.7;<br>hsa00563|Glycosylphosphatidylinositol (GPI)-anchor biosynthesis|-41.7
MyList_SUB1_MCODE_3	GO:0030497|fatty acid elongation|-24.4;GO:0042761|very long-chain fatty acid biosynthetic process|-24.4;<br>R-HSA-75876|Synthesis of very long-chain fatty acyl-CoAs|-22.1
MyList_SUB1_MCODE_4	GO:0006506|GPI anchor biosynthetic process|-15.1;GO:0006505|GPI anchor metabolic process|-15.0;GO:0097502|mannosylation|-14.8
MyList_SUB1_MCODE_5	hsa00601|Glycosphingolipid biosynthesis - lacto and neolacto series|-15.4;GO:0009247|glycolipid biosynthetic process|-13.2;<br>GO:0006664|glycolipid metabolic process|-12.4
MyList_SUB1_MCODE_6	R-HSA-5626978|TNFR1-mediated ceramide production|-15.4;R-HSA-75893|TNF signaling|-11.4;M128|PID TNF PATHWAY|-11.3
MyList_SUB1_MCODE_7	GO:0046520|sphingoid biosynthetic process|-10.0;GO:0046512|sphingosine biosynthetic process|-10.0;GO:0006670|sphingosine metabolic process|-9.5
MyList_SUB1_MCODE_8	GO:0034625|fatty acid elongation, monounsaturated fatty acid|-11.1;GO:0019368|fatty acid elongation, unsaturated fatty acid|-11.1;<br>GO:0019367|fatty acid elongation, saturated fatty acid|-11.1
MyList_SUB1_MCODE_9	GO:0089700|protein kinase D signaling|-12.7;R-HSA-1660661|Sphingolipid de novo biosynthesis|-8.5;R-HSA-428157|Sphingolipid metabolism|-7.6
MyList_SUB1_MCODE_10	WP1423|Ganglio sphingolipid metabolism|-10.2;hsa00604|Glycosphingolipid biosynthesis - ganglio series|-10.0;GO:0097503|sialylation|-9.6
